# Sib1, Sib2, and Sib3 proteins are required for ferrichrome-mediated cross-feeding interaction between *Schizosaccharomyces pombe* and *Saccharomyces cerevisiae*

**DOI:** 10.3389/fmicb.2022.962853

**Published:** 2022-07-19

**Authors:** Ariane Brault, Berthy Mbuya, Simon Labbé

**Affiliations:** Département de Biochimie et de Génomique Fonctionnelle, Faculté de Médecine et des Sciences de la Santé, Université de Sherbrooke, Sherbrooke, QC, Canada

**Keywords:** siderophore, ferrichrome, cross-feeding, fission yeast, budding yeast

## Abstract

Although *Saccharomyces cerevisiae* is unable to produce siderophores, this fungal organism can assimilate iron bound to the hydroxamate-type siderophore ferrichrome (Fc) produced and secreted by other microbes. Fc can enter *S. cerevisiae* cells *via* Arn1. Unlike *S. cerevisiae*, *Schizosaccharomyces pombe* synthesizes and secretes Fc. The *sib1*^+^ and *sib2*^+^ genes encode, respectively, a Fc synthetase and an ornithine-N^5^-oxygenase, which are required for Fc production. When both genes were expressed in *S. pombe*, cross-feeding experiments revealed that *S. cerevisiae fet3*Δ *arn1-4*Δ cells expressing Arn1 could grow in the vicinity of *S. pombe* under low-iron conditions. In contrast, deletion of *sib1*^+^ and *sib2*^+^ produced a defect in the ability of *S. pombe* to keep *S. cerevisiae* cells alive when Fc is used as the sole source of iron. Further analysis identified a gene designated *sib3*^+^ that encodes an N^5^-transacetylase required for Fc production in *S. pombe*. The *sib3*Δ mutant strain exhibited a severe growth defect in iron-poor media, and it was unable to promote Fc-dependent growth of *S. cerevisiae* cells. Microscopic analyses of *S. pombe* cells expressing a functional Sib3-GFP protein revealed that Sib3 was localized throughout the cells, with a proportion of Sib3 being colocalized with Sib1 and Sib2 within the cytosol. Collectively, these results describe the first example of a one-way cross-feeding interaction, with *S. pombe* providing Fc that enables *S. cerevisiae* to grow when Fc is used as the sole source of iron.

## Introduction

Eukaryotes require iron for their growth. Iron serves as a redox-active cofactor for numerous cellular enzymes required for essential processes such as DNA synthesis and repair, energy production, amino acid biosynthesis, and lipid metabolism (Puig et al., 2017; Galaris et al., 2019; Philpott et al., 2020). Despite the fact that iron is an abundant metal on Earth, its bioavailability is low at physiological pH because of its oxidative conversion into insoluble ferric hydroxide forms under atmospheric oxygen conditions (Aguiar et al., 2021). To overcome the challenging problem of poor iron bioavailability, all organisms, including fungi, have developed different strategies to acquire this metal ion from different sources. One strategy consists of producing siderophores or utilizing xenosiderophores, which are siderophores produced by other organisms in the same surroundings. Siderophores are low-molecular-mass molecules that specifically bind ferric iron (Haas et al., 2008; Hider and Kong, 2010; Haas, 2014). Once synthesized and secreted by producer cells, siderophores bind to iron in the external environment, and siderophore-iron complexes (holo-siderophores) can be captured by specific siderophore-mediated transport systems. These siderophore-mediated uptake systems are expressed by the producer microbe or an opportunistic microbe that is unable to produce siderophores but is capable of taking up xenosiderophore-bound iron (Miethke and Marahiel, 2007).

Under iron starvation conditions, the fission yeast *Schizosaccharomyces pombe* synthesizes, accumulates, and excretes ferrichrome (Fc), a hydroxamate-type siderophore (Schrettl et al., 2004; Mercier and Labbé, 2010). The first enzymatic step in Fc biosynthesis consists of the N^5^ hydroxylation of ornithine by an ornithine-N^5^-oxygenase, denoted Sib2 (Schrettl et al., 2004). This initial step generates N^5^-hydroxyornithine. The subsequent acetylation of N^5^-hydroxyornithine by an unidentified N^5^-transacetylase produces N^5^-acetyl-N^5^-hydroxyornithine. This substrate is then used in combination with three glycine residues to assemble the finished Fc product through the action of the non-ribosomal peptide synthetase (NRPS) Sib1 (Schrettl et al., 2004). In addition to synthesizing Fc, *S. pombe* expresses Str1, which specifically transports Fc across the plasma membrane (Pelletier et al., 2003; Plante and Labbé, 2019). In this context, after its excretion into the surrounding environment, Fc-bound iron can be recovered by *S. pombe* through its cell surface Fc transporter, Str1.

Expression of the *sib1*^+^, *sib2*^+^, and *str1*^+^ genes is regulated at the transcriptional level as a function of iron availability (Pelletier et al., 2003; Mercier and Labbé, 2010; Plante and Labbé, 2019). They are induced under conditions of iron deprivation and are repressed under iron-replete conditions. The iron-responsive transcription factor Fep1 represses *sib1*^+^, *sib2*^+^, and *str1*^+^ gene expression (Pelletier et al., 2002, 2003; Mercier and Labbé, 2010; Plante and Labbé, 2019). In contrast, when the availability of iron is limited, Fep1 is unable to bind its GATA cis-acting elements on chromatin, resulting in the transcription of *sib1*^+^, *sib2*^+^, and *str1*^+^ (Jbel et al., 2009).

Although *Saccharomyces cerevisiae* does not secrete siderophores, it possesses four genes that encode members of a subfamily of the major facilitator superfamily (MFS) of transporters that take up siderophores produced by other microbial species (Moore et al., 2003). These four genes are denoted *ARN1*, *ARN2* (*TAF1*), *ARN3* (*SIT1*), and *ARN4* (*ENB1*) (Lesuisse et al., 1998, 2001; Heymann et al., 1999, 2000a,b; Yun et al., 2000a). The four Arn1-4 transporters exhibit different siderophore specificities. Arn1 exhibits high specificity for Fc-iron, whereas Arn2 mediates triacetylfusarinine C uptake. Ferrioxamine B-iron is the preferred siderophore substrate of Arn3. A common property of Arn1, Arn2, and Arn3 is that all three transmembrane proteins transport hydroxamate-type siderophores. In contrast, Arn4 exhibits uptake activity for bacterial enterobactin, a siderophore of the catecholate class (Yun et al., 2000b; Philpott et al., 2002). The expression of *ARN1-4* allows *S. cerevisiae* to directly transport siderophores into cells under iron-deficient conditions. When siderophores are trapped and bound within the cell wall by Fit1-3 proteins (Protchenko et al., 2001; Philpott et al., 2002), *S. cerevisiae* can use its cell-surface reductases Fre1-3 to catalyze the reduction of iron bound to siderophores, fostering iron release, which in turn can be transported into the cell by the Ftr1-Fet3 permease-oxidase complex (Askwith et al., 1994; De Silva et al., 1995; Stearman et al., 1996; Askwith and Kaplan, 1998; Yun et al., 2000a,^2001^).

In microbiology, cross-feeding usually refers to an interaction in which a microbial cell utilizes a metabolite that has been produced and secreted from another cell (Pande and Kost, 2017; D’Souza et al., 2018; Fritts et al., 2021). Four criteria are considered predictive of a cross-feeding interaction. First, the process involves different species forming two genotypically or phenotypically distinct populations. Second, a metabolite is transferred from a producing cell (producer) to a neighboring cell (recipient). Third, the transferred metabolite is assimilated by the recipient. Fourth, the recipient’s ability to survive depends on its capacity to assimilate or use the transferred metabolite. There are different types of cross-feeding interactions (Seth and Taga, 2014; Pande and Kost, 2017; D’Souza et al., 2018; Fritts et al., 2021). For simplicity, the number and identity of cells that can participate in producing and/or utilizing a metabolite are often presented in a context in which only two types of microbial cells exchange a metabolite. Using this binary approach, cross-feeding has been classified as follows: (i) unidirectional or one-way cross-feeding, where a donor cell secretes a metabolite without a fitness cost that is utilized by the recipient; (ii) bidirectional or two-way cross-feeding, involving reciprocal exchange of metabolite(s) without fitness costs between two partners; (iii) one partner secretes a costly metabolite to support the recipient, which in turn supplies the producer with increased concentrations of a metabolic by-product (by-product reciprocity); and (iv) both microbial species carry the fitness cost of metabolite biosynthesis and secretion and benefit from the mutual cross-feeding interaction (also called cooperative cross-feeding) (Pande and Kost, 2017).

As secreted molecules, siderophores have been shown to participate in cross-feeding interactions in which one donor microbial strain produces and releases a siderophore that benefits another recipient microbe under iron-poor conditions (Joshi et al., 2006; Galet et al., 2015; Butaitë et al., 2017; Fritts et al., 2021). Although several examples of bacterial species are involved in siderophore cross-feeding between strains, only a limited number of yeasts exhibit such one-way cross-feeding relationships. In this study, we demonstrate that the introduction of the wild-type *ARN1* allele in *S. cerevisiae fet3*Δ *arn1-4*Δ cells restores their ability to acquire exogenous Fc. The results consistently show that *S. cerevisiae fet3*Δ *arn1-4*Δ cells expressing Arn1 are able to grow in the vicinity of *S. pombe* cells that produce Fc. Furthermore, the Fc-dependent growth of *ARN1*-expressing *S. cerevisiae fet3*Δ *arn1-4*Δ cells is improved in the presence of *S. pombe* donor cells lacking the Fc uptake transporter Str1. Inactivation of the *sib3*^+^ gene, which encodes a predicted N-transacetylase, results in the inability of *S. pombe* cells to produce Fc. *sib3*Δ mutant cells exhibit severe growth defects when inoculate in iron-poor media compared with wild-type cells. Furthermore, *S. pombe sib3*Δ cells fail to promote the growth of *S. cerevisiae fet3*Δ *arn1-4*Δ *ARN1* cells when both strains are point-inoculated in the vicinity of each other. Taken together, these results reveal that *S. pombe* cells capable of producing Fc can engage in a one-way cross-feeding interaction with *S. cerevisiae fet3*Δ *arn1-4*Δ *ARN1* cells that are unable to grow without an external Fc supply.

## Materials and methods

### Yeast strains and growth conditions

Genotypic descriptions^1^ of the *S. pombe* and *S. cerevisiae* strains used in this study are presented in Table 1. *S. pombe* strains were grown on yeast extract plus supplement (YES) medium under non-selective growth conditions, as described previously (Sabatinos and Forsburg, 2010). To transform and maintain plasmids in *S. pombe* strains, Edinburgh minimal medium (EMM) lacking a specific ribonucleotide base or particular amino acids was used for ensuring the selection of transformed yeast cells (Moreno et al., 1991). *S. cerevisiae* strains were cultured in YPD medium under non-selective conditions, whereas synthetic dropout (SC) media was used for DNA plasmid transformation, as described previously (Sherman, 2002). For cross-feeding experiments, aliquots of the indicated cultures of *S. pombe* and *S. cerevisiae* strains were spotted in the vicinity of one another on a modified synthetic dextrose minimal (SD) medium containing bacto-yeast nitrogen base (1.7 g/l), which was depleted of copper and iron, as described previously (Beaudoin and Labbé, 2001). This SD^–Cu–Fe^ medium additionally contained ammonium sulfate (5 g/L), dextrose (3%), and 225 mg/L uracil, lysine, adenine, tryptophan, and histidine.

**TABLE 1:** Yeast strains used in this study.

*S. pombe* strain	Genotype	Source
FY435	*h^+^ his7-366 leu1-32 ura4-*Δ*18 ade6-M210*	Pelletier et al., 2002
*fep1*Δ	*h^+^ his7-366 leu1-32 ura4-*Δ*18 ade6-M210 fep1*Δ*:ura4^+^*	Pelletier et al., 2002
AMY58	*h^+^ his7-366 leu1-32 ura4-*Δ*18 ade6-M210 sib1*Δ *sib2*Δ*:KAN*^r^**	Mercier and Labbé, 2010
ABY115	*h^+^ his7-366 leu1-32 ura4-*Δ*18 ade6-M210 str1*Δ*:KAN*^r^**	This study
ABY151	*h^+^ his7-366 leu1-32 ura4-*Δ*18 ade6-M210 sib2*Δ*:KAN*^r^**	This study
ABY127	*h^+^ his7-366 leu1-32 ura4-*Δ*18 ade6-M210 sib3*Δ*:KAN*^r^**	This study
ABY152	*h^+^ his7-366 leu1-32 ura4-*Δ*18 ade6-M210 sib2*Δ*:loxP fep1Δ:KAN*^r^**	This study
ABY146	*h^+^ his7-366 leu1-32 ura4-*Δ*18 ade6-M210 sib3*Δ*:loxP fep1Δ:KAN*^r^**	This study
ABY147	*h^+^ his7-366 leu1-32 ura4-*Δ*18 ade6-M210 sib1^+^-GFP:KAN*^r^**	This study
ABY148	*h^+^ his7-366 leu1-32 ura4-Δ18 ade6-M210 sib2^+^-GFP:KAN*^r^**	This study
ABY149	*h^+^ his7-366 leu1-32 ura4-Δ18 ade6-M210 sib3^+^-GFP:KAN*^r^**	This study
ABY150	*h^+^ his7-366 leu1-32 ura4-Δ18 ade6-M210 sib3^+^-Cherry:KAN*^r^**	This study
***S. cerevisiae* strain**	**Genotype**	**Source**
YPH499	*MAT***a** *ura3-52 lys2-801 ade2-101 trp1-63*Δ *his3-200*Δ *leu2-1*Δ	Yun et al., 2000a
*fet3*Δ *arn1-4*Δ	*MAT***a** *ura3-52 lys2-801 ade2-101 trp1-63*Δ *his3-200Δ leu2-1Δ fet3*Δ*:HIS3 arn1*Δ*:HISG arn2*Δ*:HISG arn3*Δ*:HISG arn4*Δ*:HISG-URA3-HISG*	Yun et al., 2000a

For detection of Fc by TLC, cells were grown in liquid cultures to an OD_600_ of 0.5 and subsequently treated with Dip (100 μM) for 5 h, unless otherwise stated. For *S. cerevisiae* growth assays on solid media, cells were spotted on SC-Leu containing 75 μM Dip or a combination of 75 μM Dip and 2 μM Fc. Growth assays for *S. pombe* strains were performed on solid media similar to that for *S. cerevisiae* cells, except that YES medium containing 0 or 140 μM Dip was used. In the case of pairwise co-culture assays, iron-replete *S. pombe* cells grown to an OD_600_ of 1.0 were subsequently adjusted to 1 × 10^7^ cells/10 μl and then spotted on SD^–Cu–Fe^ medium. In parallel experiments, mid-logarithmic phase cultures of *S. cerevisiae fet3*Δ *arn1-4*Δ cells expressing *ARN1* were diluted (3,000 cells/10 μL) and spotted in the vicinity of *S. pombe* strains. In the case of pairwise liquid co-culture assays, *S. pombe* strains were precultured in the presence of 10 μM FeCl_3_, whereas *S. cerevisiae* strains were grown in liquid SD^–Cu–Fe^ medium. Subsequently, the indicated *S. pombe* and *S. cerevisiae* strains were mixed in a 1:1 ratio and grown in liquid SD^–Cu–Fe^ medium in the presence of Dip (100 μM) for 18 h. Each pairwise co-culture was examined using Nomarski optics for determining the number of *S. cerevisiae* cells vs. the number of *S. pombe* cells during this period. To monitor the mRNA and protein steady-state levels of Str1 and Sib1-3 in response to changes in iron levels, *S. pombe* cultures were seeded to an OD_600_ of 0.5, grown to the exponential phase (OD_600_ of 1.0) and either left untreated or treated with Dip (250 μM) or FeCl_3_ (100 μM) for 90 min, as described previously (Mercier et al., 2008).

### Plasmids

The *S. cerevisiae ARN1* coding sequence was isolated by PCR and cloned into the *Spe*I-*Xma*I-cut p415GPD vector (Mumberg et al., 1995). The resulting centromeric plasmid was named p415GPD*ARN1*. We amplified the *ARN1* gene without its stop codon, using primers containing *Spe*I and *Xma*I restriction sites. The resulting PCR product was inserted into the corresponding sites of p415GPD to create p415GPD*ARN1*nostop. The coding region of GFP was amplified by PCR, digested with *Xma*I and *Xho*I, and inserted in-frame with *ARN1* into the corresponding sites of p415GPD*ARN1*nostop, generating the p415*GPDARN1-GFP* construct. The *S. pombe str1*^+^ gene was isolated by PCR using primers amplifying the *str1*^+^ locus starting at -966 bp from the initiator codon up to the stop codon of *str1*^+^. The PCR product was digested with *Kpn*I and *Bam*HI, and subsequently inserted into the corresponding sites of pJK148 (Keeney and Boeke, 1994). The resulting plasmid was denoted pJK-966*str1*^+^. The integrative pJK-966*str1^+^-GFP* plasmid was constructed as previously described (Plante and Labbé, 2019). To generate the integrative pJK-501*sib3*^+^ plasmid, a 1,506-bp *Apa*I-*Sac*I-amplified DNA segment containing the *sib3*^+^ gene starting at position -501 from the start codon up to the stop codon was cloned into the *Apa*I and *Sac*I sites of pJK148. The same *sib3*^+^ DNA segment was amplified without its stop codon and then cloned into the *Apa*I and *Xma*I sites of pJK148, creating the plasmid pJKsib3nostop. Next, the *GFP*, *Cherry*, or *TAP* coding sequence was inserted into pJKsib3nostop at the *Xma*I and *Sac*I sites, generating three plasmids, denoted pJK*sib3^+^-GFP*, pJK*sib3^+^-Cherry*, and pJK*sib3^+^-TAP*, respectively. For generating the plasmid used for pull-down experiments, the *sib3*^+^ locus starting at –501 bp from the start codon up to the penultimate codon of the gene was isolated by PCR using primers containing *Apa*I and *Xma*I restriction sites. The PCR-amplified DNA fragment was cloned into the *Apa*I and *Xma*I sites of pSP1 (Cottarel et al., 1993), generating the plasmid pSP1sib3nostop. Subsequently, the coding sequence of NTAP was isolated by PCR, digested with *Xma*I and *Sac*I, and inserted in-frame with *sib3*^+^ into pSP1sib3nostop, creating the plasmid pSP1sib3-TAP.

The 5′ end of the *sib1*^+^- *sib2*^+^ intergenic promoter region containing 597 bp was PCR-amplified using a set of primers that generated *Apa*I and *Pst*I sites at the extremities of the PCR product. The intergenic promoter fragment was cloned into the corresponding sites of pJK148 or pJB1, creating pJK-597prom or pJB1-597prom plasmids. The *sib2*^+^ coding sequence was amplified without its stop codon and inserted downstream of the intergenic promoter region using *Pst*I and *Xma*I restriction sites. Subsequently, the GFP coding sequence was inserted downstream and in-frame to the *sib2*^+^ ORF at the *Xma*I and *Sac*I restriction sites, creating pJK-597*sib2^+^-GFP* and pJB1-597*sib2^+^-GFP* plasmids, respectively.

The plasmid pJB1tpsib2-GFP was constructed as follows: an *Apa*I-*Eco*RV PCR-amplified fragment from the *tpx1*^+^ promoter containing the first 1,500 bp of the 5′-non-coding region was inserted into the *Apa*I and *Eco*RV sites of pJB1, creating pJB1-1500tpx1prom. The *sib2*^+^ ORF without its stop codon was isolated by PCR using primers containing *Eco*RV and *Xma*I restriction sites. The PCR product was cloned into the corresponding sites of pJB1-1500tpx1prom, creating the plasmid pJB1tpsib2nostop. A DNA sequence encoding GFP was amplified using primers containing the *Xma*I and *Sac*I restriction sites, and the resulting PCR product was digested and inserted into pJB1tpsib2nostop.

For creating strains in which the GFP or Cherry coding sequence was integrated downstream of and in-frame to the 3′ chromosomal regions of *sib1*^+^, *sib2*^+^, and *sib3*^+^ genes, a PCR-based gene fusion strategy using the pFA6a-GFP(S65T)-kanMX6 or pFA6A-Cherry-kanMX6 module was performed as described previously (Bahler et al., 1998). This approach allowed site-specific integration of GFP or Cherry at the chromosomal locus of *sib1*^+^, *sib2*^+^, or *sib3*^+^, fostering the replacement of the wild-type allele with GFP- or Cherry-tagged *sib1*^+^, *sib2*^+^, or *sib3*^+^ alleles.

### RNA extraction and analysis by real-time quantitative reverse transcription PCR assays

Total RNA was isolated from the indicated cell cultures using the hot phenol method, as described previously (Chen et al., 2003). Reverse transcription reactions were performed in a 20-μL reaction mixture that contained 1 μg of RNA, 2 μL of random primer mix (60 μM) [including 25 μM oligo(dT) and 35 μM random hexamers], 2 μL of 10 × RT buffer, 1 μL of dNTP mix (10 mM), 0.2 μL of RNase inhibitor (40 U/μL), and 0.2 μL of MMuLV RT (200 U/μL). cDNA synthesis was performed using the following steps: hybridization for 5 min at 25°C, elongation for 60 min at 42°C, and inactivation for 20 min at 65°C. qPCR reactions were performed in a 20-μL reaction mix containing 2 μL of cDNA (1:10 dilution), 300 nM of forward and reverse specific primers, and 10 μL of Supermix qPCR 2X that included SYBR Green, dNTPs, and thermostable DNA polymerase. The reaction was performed on a CFX96 Touch Real-Time PCR System (Bio-Rad) with the following steps: 3 min at 95°C (initial denaturation), 15 s at 95°C, 30 s at 60°C, and 30 s at 72°C. The last three steps were repeated for over 45 cycles. Each target gene (*sib1*^+^, *sib2*^+^, or *sib3*^+^) was analyzed in experiments that included a minimum of three biological replicates, and assays were performed in triplicate. Results were considered valid if the target-specific fluorescent signal showed a C_*t*_ value ≤ 37 cycles, and all positive and negative control reactions yielded successful and no amplification, respectively. Fold changes of each transcript (*sib1*^+^, *sib2*^+^, or *sib3*^+^) in wild-type and *fep1*Δ mutant samples were calculated using the ΔΔCt method normalized to *act1*^+^, the internal control (Livak and Schmittgen, 2001; Schmittgen and Livak, 2008; Protacio et al., 2022). Calculations were performed using the following equation: ΔΔCt = [(Ct gene–Ct ref) in wild-type] vs. [(Ct gene–Ct ref) in *fep1*Δ) under the indicated experimental conditions that were performed as a function of iron availability. In the case of *sib1*^+^, the primer pair allowed the detection of an amplicon corresponding to the coding region between positions + 810 and + 910 down to the first nucleotide of the initiator codon. For *sib2*^+^ and *sib3*^+^, the amplicons corresponded to the coding regions between positions + 205 to + 301 and + 362 to + 451, respectively. To detect the expression of *act1*^+^, a primer pair was used for amplifying the coding sequence between + 173 and + 280 down to the first base of the ATG codon of *act1*^+^.

### Detection of Fc

A modified Fc extraction method (Moore et al., 2003; Mercier and Labbé, 2010; Plante and Labbé, 2019) was used for analyzing the presence of Fc in *S. cerevisiae* and *S. pombe* strains. Yeast cells were harvested at the exponential growth phase and resuspended in saturated ammonium sulfate solution (300 μL; 571 g/L) containing 1.6 mM FeCl_3_. After the addition of benzyl alcohol (Sigma-Aldrich, #305197) and glass beads (200 μL) to the resuspended cells, the mixtures were lysed using FastPrep disruption (MP-24 instrument; MP Biomedicals). Cell lysates were mixed with an additional 300 μL of saturated ammonium sulfate solution. After mixing, the samples were centrifuged (13,000 rpm for 10 min at 25°C) and the organic layers (upper fraction) were collected. The latter fraction was diluted with 3 volumes (1.2 mL) of diethyl ether (Acros Organics, #364335000) and mixed by vortexing with water (150 μL). The phases were separated by centrifugation (13,000 rpm for 10 min), and the aqueous layer (lower fraction) was collected, washed with 1 vol of diethyl ether, and lyophilized. Dried samples were resuspended in 3 μL water and spotted on preheated silica gel 60 F_254_ thin-layer chromatography plastic sheets (EMD Millipore). TLC was performed using a solvent containing 80% aqueous methanol. Commercially purified holo-Fc (15 μg) (Sigma-Aldrich, F8014) was used as the positive control for signal detection.

### Fluorescence microscopy and protein analysis

Fluorescence and differential interference contrast images (Nomarski) of cells were obtained using a Nikon Eclipse E800 epifluorescence microscope (Nikon, Melville, NY) equipped with a Hamamatsu ORCA-ER digital cooled camera (Hamamatsu, Bridgewater, NJ). The cells were viewed using 1,000 × magnification and the following two filters: 465–495 nm (GFP signal) and 510–560 nm (Cherry signal). Representative fields of cells shown correspond to a minimum of three independent experiments. Furthermore, the cell fields shown represent protein localization in 200 cells tested per condition.

Cell extracts from *S. cerevisiae* were prepared with glass beads using a FastPrep-24 instrument (MP Biomedicals, Solon, OH). Cells were lysed in TMN_150_ buffer containing 50 mM Tris-HCl (pH 7.5), 150 mM NaCl, 5 mM MgCl_2_, 1% Nonidet P-40, 1 mM phenylmethylsulfonyl fluoride (PMSF), and a complete protease inhibitor cocktail (P8340, Sigma-Aldrich). Cell lysates were incubated with Triton X-100 (1%) for 30 min on ice prior to being resolved on 7% sodium dodecyl sulfate (SDS)-polyacrylamide gels. Arn1-GFP and PGK proteins were detected by immunoblotting with anti-GFP and anti-PGK antibodies, respectively. For *S. pombe* Str1 protein detection, cells were lysed in Thorner buffer [40 mM Tris-HCl (pH 6.8), 8 M urea, 5% SDS, 0.01 mM ethylenediaminetetraacetic acid (EDTA), 1% β-mercaptoethanol and 0.4 mg/mL bromophenol blue] with glass beads using two successive rounds of disruption using the FastPrep-24 instrument. Between each round, cell lysates were incubated for 10 min at 37°C and centrifuged to retrieve the supernatant fraction. After 15 min at 37°C, aliquots of the supernatant fraction were resolved by electrophoresis on 8% SDS-polyacrylamide gels prior to Western blot analysis. To analyze Sib2 and Sib3 protein levels, whole cell extracts were prepared using a trichloroacetic acid (TCA) extraction method as described previously (Foiani et al., 1994; Ioannoni et al., 2012).

In pull-down experiments, *sib2*Δ *sib3*Δ cells were co-transformed with pSP1sib3-TAP and pJB1tpsib2-GFP or pSP1sib3-TAP and pBPGFP. Cultures were grown in EMM to an OD_600_ of 1.0 in the presence of Dip (50 μM). After washes, aliquots of the cultures were left untreated or treated with Dip (250 μM) or FeCl_3_ (100 μM) for 90 min. Total cell lysates were prepared by glass bead disruption and subjected to pull-down assays using IgG-Sepharose 6 Fast-Flow beads (GE Healthcare) as described previously (Jacques et al., 2014).

Immunodetection of GFP-tagged proteins (Arn1-GFP, Str1-GFP, Sib2-GFP, and Sib3-GFP), TAP-tagged Sib3, PGK, and α-tubulin was performed using the following primary antibodies: monoclonal anti-GFP antibody B-2 (Santa Cruz Biotechnology), polyclonal anti-mouse IgG antibody (ICN Biomedicals), monoclonal anti-PGK antibody 22C5-D8 (Molecular Probes), and monoclonal anti-α-tubulin antibody B-5-1-2 (Sigma-Aldrich). After incubation with the primary antibodies, the membranes were washed and incubated with the appropriate horse-radish peroxidase-conjugated secondary antibodies (Amersham Biosciences). Proteins were detected using ECL reagents (Amersham Biosciences) and visualized using chemiluminescence.

## Results

### Expression of Arn1 in *Saccharomyces cerevisiae fet3Δ arn1-4*Δ cells restores their ability to acquire ferrichrome

**FIGURE 1:**
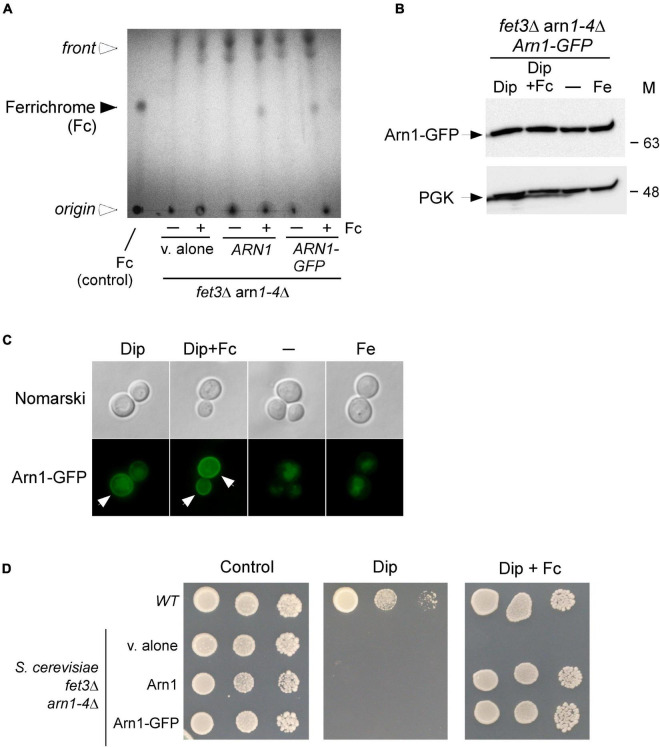
Expression of ARN1 in *S. cerevisiae fet3Δ arn1-4*Δ cells complements the Fc acquisition deficiency of cells defective in the uptake of Fc. **(A)** *S. cerevisiae fet3*Δ *arn1-4*Δ mutant cells expressing an empty plasmid, *ARN1* or *ARN1-GFP* alleles were grown in SC medium to the mid-logarithmic phase. The cells were then incubated in the presence of Dip (100 μM) without Fc supplementation or with Fc supplementation (2 μM) for 5 h. Whole extracts were prepared and total Fc content was analyzed by thin-layer chromatography on silica gel sheets. Commercially purified Fc (15 μg) was run as a reference (control, far left lane). The solid arrowhead indicates the migration position of Fc, whereas the open arrowheads show the position of sample loading and the front of gel migration. **(B)** *S. cerevisiae fet3*Δ *arn1-4*Δ cells expressing Arn1-GFP were grown to the mid-logarithmic phase and subsequently left untreated (-) or treated with Dip (100 μM), Dip plus Fc (2 μM), or iron (Fe, 100 μM) for 5 h. Whole cell extract preparations were analyzed using immunoblot assays with anti-GFP and anti-PGK antibodies. The positions of the molecular weight standards (in kDa) are indicated on the right side. **(C)** Aliquots of cultures used in *panel B* were analyzed by fluorescence microscopy for visualizing cellular location of Arn1-GFP. Cell morphology was examined using Nomarski optics. White arrows indicate the cell periphery. The results are representative of three independent experiments. **(D)** Aliquots of cultures used in *panel A* were spotted in serial dilutions onto medium without Dip or Fc supplementation (control) or supplemented with Dip or a combination of Dip (75 μM) and Fc (2 μM). An isogenic wild-type (WT) strain was grown under the same conditions used in **(A)** and then spotted at different cellular densities onto the same indicated solid media as a control. All plates were incubated for 4 days at 30°C, and photographed.

Previous studies have shown that the Arn1 protein in *S. cerevisiae* functions as an Fc transporter (Heymann et al., 2000b; Yun et al., 2000b; Lesuisse et al., 2001). To validate the role of Arn1 in the context of our experimental system, we used an *S. cerevisiae fet3*Δ *arn1-4*Δ mutant strain defective in the uptake of siderophore iron. To this strain, a wild-type *ARN1* allele expressed from a centromeric plasmid was returned by transformation. Similarly, a *GFP*-tagged *ARN1* allele was returned by transformation to determine whether the fusion allele retained its wild-type function. *fet3*Δ *arn1-4*Δ cells expressing an empty plasmid, *ARN1*, or *ARN1-GFP* allele were grown to the mid-logarithmic phase and then incubated in the presence of the iron chelator Dip (100 μM) without Fc supplementation or with Fc supplementation (2 μM) for 5 h. To determine whether the cells accumulated Fc when *ARN1* or *ARN1-GFP* was reintroduced into *fet3*Δ *arn1-4*Δ cells, extracts from the indicated transformed strains were analyzed by thin-layer chromatography (TLC). In Fc-treated *fet3*Δ *arn1-4*Δ cells expressing *ARN1* or *ARN1-GFP*, a positive Fc signal was detected, indicating their ability to assimilate exogenous Fc (Figure 1A). In contrast, in the absence of exogenous Fc, the same strains failed to show an Fc signal (Figure 1A). Extracts from the *fet3*Δ *arn1-4*Δ mutant strain harboring an empty plasmid were devoid of detectable Fc, irrespective of the absence or presence of exogenous Fc into the medium (Figure 1A).

In the case of *fet3*Δ *arn1-4*Δ cells that were transformed with a GFP epitope-tagged *ARN1* allele under the control of the constitutive *GDP* promoter, steady-state protein levels of Arn1-GFP were analyzed by immunoblotting. The results showed that Arn1-GFP was produced under all experimental conditions, including basal, iron-starved, and iron-replete conditions in the presence or absence of Fc (Figure 1B). Considering that Arn1 was required for the uptake of exogenous Fc, we next sought to examine its localization when *fet3*Δ *arn1-4*Δ cells expressing *ARN1-GFP* were incubated under low-iron conditions in the presence of exogenous Fc. After 5 h, the Arn1-GFP fluorescence signal was primarily detected at the contour of the cells (Figure 1C). When *fet3*Δ *arn1-4*Δ cells expressing *ARN1-GFP* were cultured in Fc-free medium containing Dip (100 μM), analysis of the localization of Arn1-GFP revealed that only a fraction of the fluorescence signal was observed at the periphery of the cells, whereas a significant proportion of the Arn1-GFP signal appeared as fluorescent intracellular structures within the cell (Figure 1C). Under basal and iron-replete conditions, fluorescence microscopy revealed that Arn1-GFP signal was mainly detected in the cytosol of the cells, without distinct fluorescence visible at the periphery of the cells (Figure 1C).

Consistent with Arn1 functioning as an Fc-iron transporter, the results showed that when *ARN1* and *ARN1-GFP* alleles were returned in *fet3*Δ *arn1-4*Δ cells, their expression restored growth in the presence of exogenous Fc (2 μM) under low-iron conditions (Figure 1D). Growth rescue was Fc-specific because utilization of media without Fc supplementation resulted in no detectable growth under iron-limited conditions (Dip, 75 μM) (Figure 1D). An isogenic wild-type strain could grow on synthetic complete medium that was left untreated (control) or supplemented with Dip (75 μM) or Dip plus Fc (2 μM) (Figure 1D). Collectively, these results showed that Arn1 is required for *S. cerevisiae* growth when cells take up exogenous Fc as the sole source of iron under iron-limited conditions.

### Cross-feeding between Fc-producing *Schizosaccharomyces pombe* cells and *Saccharomyces cerevisiae fet3Δ arn1-4*Δ cells expressing ARN1

**FIGURE 2:**
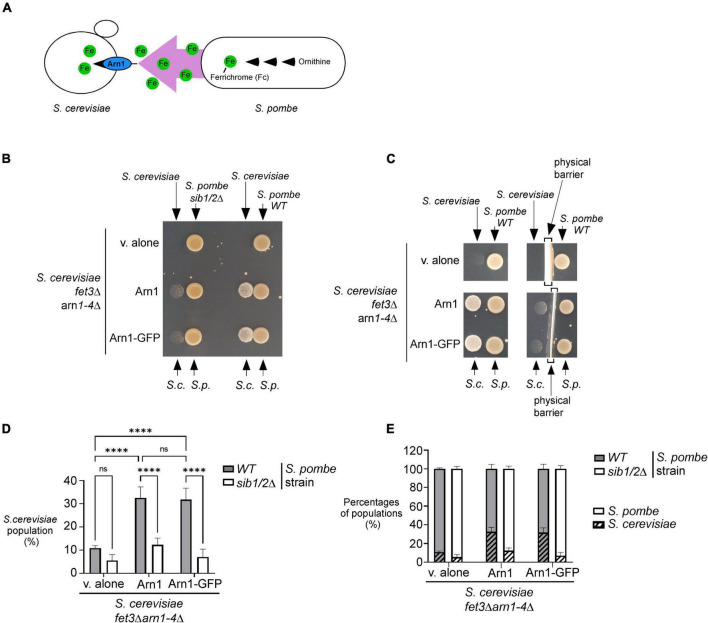
*S. pombe* Fc promotes growth of a *S. cerevisiae fet3*Δ *arn1-4*Δ mutant in which the *ARN1* gene is returned. **(A)** Schematic representation of a *S. cerevisiae fet3*Δ *arn1-4*Δ mutant expressing *ARN1* in co-culture with a *S. pombe* strain that was competent to synthesize and excrete Fc-iron. **(B,C)** *S. pombe* wild-type (WT) and *sib1*Δ *sib2*Δ strains were grown to an OD_600_ of 1.0 in the presence of FeCl_3_ (10 μM), and half of cells (1 × 10^7^ cells/10 μl) were spotted (right side of each pair of spots) onto SD^–Cu–Fe^ medium. *S. cerevisiae fet3*Δ *arn1-4*Δ cells harboring an empty vector (v. alone) or expressing *ARN1* or *ARN1-GFP* were grown in SD^–Cu–Fe^ to an OD_600_ of 1.0. Cells were washed, diluted 10,000-fold, and spotted (3,000 cells/10 μL) (left side of each pair of spots) onto SD^–Cu–Fe^ medium in the vicinity of the *S. pombe* strains. In the representative experiment in **(C)** (right side), a physical barrier was installed between point-inoculated *S. cerevisiae* and *S. pombe* strains. S.c., *S. cerevisiae*; S.p., *S. pombe*. **(D,E)** *S. pombe* wild-type (WT) and *sib1*Δ *sib2*Δ strains were mixed with the indicated plasmid-transformed *S. cerevisiae fet3*Δ *arn1-4*Δ strain in a 1:1 ratio. Each pairwise co-culture was grown in liquid SD^–Cu–Fe^ medium in the presence of Dip (100 μM) for 18 h. The morphology of yeast cells was examined by Nomarski optics for determining the number of *S. cerevisiae* cells vs. the total number of fungal cells. Representative results are presented for the percentages of *S. cerevisiae* cells with the indicated plasmids in co-culture with *S. pombe* (WT or *sib1*Δ *sib2*Δ) **(D)**. Comparative results of percentages of each population representing *S. cerevisiae* cells vs. *S. pombe* cells after they were grown together for 18 h **(E)**. A minimum of 300 cells were examined for each pairwise co-culture. The results are representative of three independent experiments. The error bars indicate the standard deviation (± SD; error bars). The asterisks correspond to *p* < 0.0001 (****) (two-way ANOVA with Tuckey’s multiple comparisons test against the genotypes of *S. pombe* and *S. cerevisiae* strains), whereas ns stands for not significant.

Previous studies have shown that *S. pombe* biosynthesizes, accumulates and secretes Fc, especially under conditions of iron deprivation (Schrettl et al., 2004; Mercier and Labbé, 2010; Plante and Labbé, 2019). Considering that *S. cerevisiae* lacks the ability to synthesize siderophores but can assimilate exogenous siderophores, we examined whether *S. pombe* could support the growth of *S. cerevisiae* through its ability to donate Fc-iron as a sole source of iron (Figure 2A). For testing this possibility, an *S. pombe* wild-type or *sib1*Δ *sib2*Δ strain was cultured in the presence of FeCl_3_ (10 μM) and subsequently spotted (1 × 10^7^ cells/10 μl) on a copper- and iron-poor medium, denoted SD^–Cu–Fe^. The *S. cerevisiae fet3*Δ *arn1-4*Δ mutant strain that had been transformed with an empty vector, an untagged *ARN1* or GFP-tagged *ARN1* allele was used for cross-feeding assays. *S. cerevisiae* cultures were grown in SD^–Cu–Fe^ medium until the logarithmic phase. At this point, the indicated cultures were diluted 10,000-fold and point-inoculated in the vicinity of *S. pombe* cells (wild-type and *sib1*Δ *sib2*Δ strains). The results showed that the *S. cerevisiae fet3*Δ *arn1-4*Δ cells expressing *ARN1* and *ARN1-GFP* alleles were able to grow in the vicinity of the wild-type *S. pombe* strain (Figure 2B). In contrast, *S. cerevisiae fet3*Δ *arn1-4*Δ cells were unable to grow in the presence of an *S. pombe sib1*Δ *sib2*Δ mutant strain defective in Fc biosynthesis, regardless of the absence or presence of *ARN1* and *ARN1-GFP* alleles (Figure 2B). Considering that Fc is a small molecule secreted by the donor *S. pombe*, we tested whether physical separation using a sterile spacer between *S. pombe* and *S. cerevisiae* prevented cross-feeding. Indeed, the presence of a physical barrier that blocked the diffusion of *S. pombe* Fc in the surrounding medium resulted in no significant growth of *S. cerevisiae* cells containing an untagged *ARN1* or GFP-tagged *ARN1* allele (Figure 2C). In contrast, *S. cerevisiae fet3*Δ *arn1-4*Δ cells expressing *ARN1* and *ARN1-GFP* alleles grew on plates devoid of a spacer that served as a physical barrier (Figure 2C). This result suggests that Fc dispersed in the medium can reach neighboring cells of *S. cerevisiae* that can benefit from the Fc available in their proximal environment.

To further analyze the effects of cross-feeding between the two species of yeast, budding and fission yeast forms were examined using Nomarski optics for comparing the proportion of *S. cerevisiae fet3*Δ *arn1-4*Δ cells with the proportion of *S. pombe* cells (wild-type or *sib1*Δ *sib2*Δ strain) when both yeast species were co-cultured in SD^–Cu–Fe^ liquid medium over 18 h. After mixing the indicated cultures of *S. cerevisiae* and *S. pombe* (wild-type strain) in a 1:1 ratio, microscopic analysis of co-culture aliquots showed that *S. cerevisiae fet3*Δ *arn1-4*Δ cells harboring an empty plasmid exhibited 21.8 and 21.0% less growth compared to *S. cerevisiae fet3*Δ *arn1-4*Δ cells expressing *ARN1* and *ARN1-GFP* alleles, respectively, over a period of 18 h (Figure 2D). Interestingly, this difference in their ability to grow was markedly attenuated when *S. cerevisiae fet3*Δ *arn1-4*Δ cells were co-cultured with *S. pombe sib1*Δ *sib2*Δ cells defective in Fc biosynthesis. Over the same period (18 h), when they were co-cultured with *S. pombe sib1*Δ *sib2*Δ cells, *S. cerevisiae fet3*Δ *arn1-4*Δ cells expressing *ARN1* and those expressing *ARN1-GFP* alleles exhibited 20.3 and 24.8% less growth, respectively, compared to when they were grown in the presence of Fc-producing *S. pombe* cells (wild-type) (Figure 2D). *ARN1*- or *ARN1-GFP*-expressing *S. cerevisiae* strains were co-cultured with *S. pombe* (wild-type or *sib1*Δ *sib2*Δ) strains. The results showed that growth of *S. cerevisiae* cells was very limited when they were co-cultured with *S. pombe sib1*Δ *sib2*Δ cells because of the lack of Fc production (from *S. pombe*) to fuel the growth of *S. cerevisiae* cells (Figure 2E). Taken together, these results indicate that Fc produced by *S. pombe* promotes the growth of *S. cerevisiae fet3*Δ *arn1-4*Δ cells expressing the Fc transporter Arn1, when Fc-bound iron is the sole source of iron.

### Fc-dependent growth of *Saccharomyces cerevisiae fet3Δ arn1-4*Δ cells expressing Arn1 is improved in the presence of *Schizosaccharomyces pombe* cells lacking Str1

**FIGURE 3:**
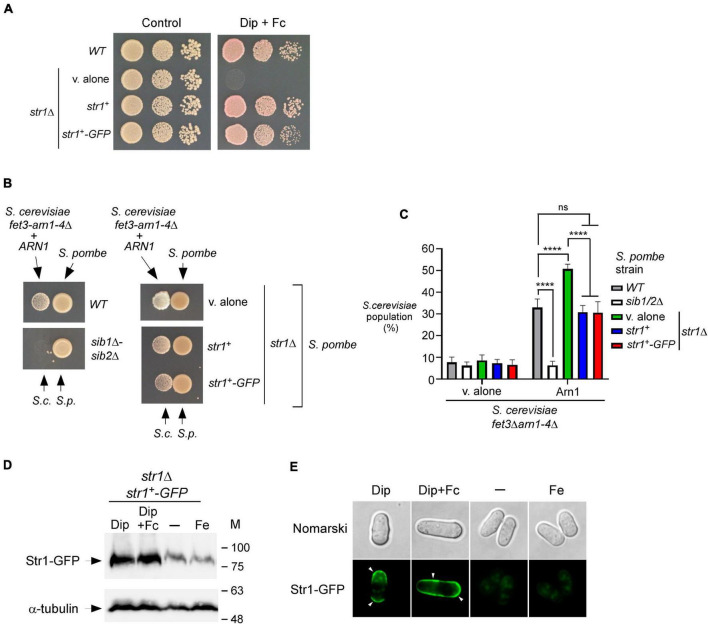
*str1*^+^ gene disruption in *S. pombe* favors Fc-dependent growth of an *S. cerevisiae fet3*Δ *arn1-4*Δ mutant expressing *ARN1*. **(A)** *S. pombe str1*Δ strain containing an empty vector (v. alone) or expressing *str1*^+^ and *str1^+^-GFP* alleles were grown to an OD_600_ of 1.0. Cells were spotted in serial dilutions onto YES medium containing Dip (140 μM) and Fc (2 μM) (right side). Cell viability of *S. pombe* wild-type (WT) and *str1*Δ strains was assayed on untreated medium (control, left side). **(B)** *S. pombe* wild-type, *sib1*Δ *sib2*Δ, and *str1*Δ strains were grown to an OD_600_ of 1.0 under iron-replete conditions (10 μM FeCl_3_). *str1*Δ cells contained an empty vector (v. alone) or expressed either the *str1*^+^ or *str1^+^-GFP* allele. At this point, the cells were spotted (1 × 10^7^ cells/10 μl) (right side of each pair of spots) onto SD^–Cu–Fe^ medium. *S. cerevisiae fet3*Δ *arn1-4*Δ cells expressing *ARN1* were grown to an OD_600_ of 1.0. At this stage, the cells were diluted 10,000-fold and spotted (3,000 cells/10 μL) in the vicinity of the *S. pombe* strains (left side of each pair of spots). S.c., *S. cerevisiae*; S.p., *S. pombe*. **(C)** The indicated *S. pombe* strains were mixed with the plasmid-transformed *S. cerevisiae fet3*Δ *arn1-4*Δ strain (harboring an empty plasmid or *ARN1* allele) in a 1:1 ratio. Each pairwise co-culture was grown in liquid SD^–Cu–Fe^ medium in the presence of Dip (100 μM) for 18 h. Morphology of yeast cells was examined by Nomarski optics for determining the number of *S. cerevisiae* cells vs. the total number of fungal cells. Representative results are shown for the percentages of *S. cerevisiae* cells in co-culture with *S. pombe* (WT, *sib1*Δ *sib2*Δ or *str1*Δ containing an empty plasmid, *str1*^+^, or *str1^+^-GFP* allele). A minimum of 300 cells were examined for each pairwise co-culture. The results are representative of three independent experiments. Error bars indicate standard deviation (± SD; error bars). The asterisks correspond to *p* < 0.0001 (****) (two-way ANOVA with Tukey’s multiple comparisons test against the indicated *S. pombe* and *S. cerevisiae fet3*Δ *arn1-4*Δ *ARN1* strains), whereas ns stands for not significant. **(D)** *str1*Δ cells expressing Str1-GFP were left untreated (-) or treated with FeCl_3_ (Fe, 100 μM), Dip (100 μM), or Dip plus Fc (2 μM) for 90 min. Aliquots of total cell extract preparations were analyzed using immunoblot assays with anti-GFP and anti-α-tubulin antibodies. The positions of the molecular weight standards (in kDa) are indicated on the right side. **(E)** Fluorescence microscopy was performed on cells incubated from each group of cultures described in **(D)** to visualize cellular location of Str1-GFP. Cell morphology was examined using Nomarski optics. White arrowheads indicate the cell periphery. Results are representative of three independent experiments.

Previous studies have shown that Fc assimilation relies on Str1 in *S. pombe* (Pelletier et al., 2003; Plante and Labbé, 2019). To further validate that *S. pombe* cells require the presence of Str1 for the acquisition of exogenous Fc in the context of the current experimental system, we used a *str1*Δ mutant strain in which an empty vector, *str1*^+^ allele, or *str1^+^-GFP* allele was returned by integration. Proliferating *str1*Δ cells bearing *str1*^+^, *str1^+^-GFP*, or an empty vector were spotted onto YES medium without Dip supplementation (control) or supplemented with a combination of Dip (140 μM) and Fc (2 μM). The cells carrying a disrupted *str1*^+^ allele (*str1*Δ containing an empty plasmid) failed to grow under low-iron conditions when exogenous Fc-bound iron was added as the sole source of iron (Figure 3A). In contrast, under these conditions, *str1*Δ cells expressing *str1*^+^ or *str1^+^-GFP* allele exhibited robust growth (Figure 3A). As the control, the wild-type strain was able to grow on YES medium without Dip supplementation or with Dip (140 μM) and Fc (2 μM) supplementation (Figure 3A).

Considering the property of Str1 to take up Fc-iron in *S. pombe*, we reasoned that its inactivation (*str1*Δ) could benefit the growth of *ARN1*-expressing *S. cerevisiae fet3*Δ *arn1-4*Δ cells in co-culture experiments, since *S. pombe str1*Δ donor cells were unable to retrieve the Fc that they produced. To test this possibility, a *str1*Δ strain that had been transformed with an empty vector, an untagged *str1*^+^ allele, or GFP-tagged *str1*^+^ allele was precultured under iron-replete conditions (10 μM FeCl_3_) and subsequently spotted on the SD^–Cu–Fe^ medium. *S. cerevisiae fet3*Δ *arn1-4*Δ cells expressing *ARN1* were grown to the logarithmic phase in SD^–Cu–Fe^ liquid medium, diluted, and point-inoculated in the vicinity of *str1*Δ cells. The growth of recipient *S. cerevisiae fet3*Δ *arn1-4*Δ *ARN1* cells was more robust near *S. pombe str1*Δ cells harboring an empty plasmid than near *S. pombe str1*Δ cells expressing functional *str1*^+^ and *str1^+^-GFP* alleles (Figure 3B). As the negative control, the recipient *S. cerevisiae fet3*Δ *arn1-4*Δ *ARN1* cells were unable to grow when spotted in the vicinity of *S. pombe sib1*Δ *sib2*Δ cells, which are defective in Fc production (Figure 3B).

Additional co-culture liquid assays were performed for verifying that *S. pombe str1*Δ cells were indeed more effective than Str1-expressing cells in promoting cross-feeding of *S. cerevisiae fet3*Δ *arn1-4*Δ *ARN1* cells. Prior to the co-cultures of *S. pombe* and *S. cerevisiae*, *S. pombe* strains were separately grown to an OD_600_ of 1.0 in SD^–Cu–Fe^ medium supplemented with FeCl_3_ (10 μM), whereas *S. cerevisiae fet3*Δ *arn1-4*Δ *ARN1* cells were separately grown in the same medium without FeCl_3_ supplementation. Following precultures, *S. pombe* and *S. cerevisiae* strains were diluted, and the co-cultures were initiated at a 1:1 ratio and monitored by microscopic examination. *S. cerevisiae fet3*Δ *arn1-4*Δ *ARN1* cells exhibited 17.8–20.1% more growth when co-cultured with *S. pombe str1*Δ cells compared to wild-type (*str1*^+^) or *str1*Δ cells expressing *str1*^+^ and *str1^+^-GFP* alleles (Figure 3C). As the negative control, *S. cerevisiae fet3*Δ *arn1-4*Δ *ARN1* cells exhibited poor growth when co-cultured with *S. pombe sib1*Δ *sib2*Δ cells (Figure 3C). This inability to grow was comparable to the background growth level of *S. cerevisiae fet3*Δ *arn1-4*Δ cells lacking Arn1, which are defective in taking up Fc produced by prototrophic *S. pombe* strains (Figure 3C).

Previous studies in *S. pombe* have shown that transcript levels of *str1*^+^ are induced in response to iron deficiency (Pelletier et al., 2003; Plante and Labbé, 2019). In co-culture experiments, the growth of the recipient *S. cerevisiae fet3*Δ *arn1-4*Δ *ARN1* strain was dependent on Fc production by *S. pombe* cells that secreted Fc into their external environment under low-iron conditions. Using this biological system and immunoblotting assays, we tested whether the protein levels of Str1 were increased when *S. pombe* cells harboring the *str1^+^-GFP* allele were grown in SD^–Cu–Fe^ medium in the presence of Dip (100 μM) or a combination of Dip and Fc (2 μM) (Figure 3D). In cells expressing *str1^+^-GFP* that were left untreated or incubated in the presence of iron (100 μM), the levels of Str1-GFP decreased compared with the protein levels of Str1-GFP observed in Dip-treated cells (Figure 3D). Fluorescence microscopy analysis consistently revealed that Str1-GFP was primarily localized at the cell surface when Str1-GFP-expressing *str1*Δ cells were incubated with Dip (100 μM) (Figure 3E). When the Dip-treated cells were supplemented with Fc (2 μM), the green fluorescence signal associated with Str1-GFP was stronger at the contour of the cell (Figure 3E). In contrast, the Str1-GFP fluorescence signal was dramatically reduced when the cells were left untreated or treated with iron (100 μM) (Figure 3E). Under these conditions, Str1-GFP fluorescence was observed as a weakly fluorescent intracellular structure within the cell (Figure 3E). Taken together, these results show that *str1*Δ disruption, which abolishes the capacity of *S. pombe* cells to assimilate exogenous Fc, favors the Fc-dependent growth of an *S. cerevisiae fet3*Δ *arn1-4*Δ mutant expressing *ARN1*.

### Disruption of sib3^+^ leads to cross-feeding inhibition between *Schizosaccharomyces pombe* and *Saccharomyces cerevisiae fet3Δ arn1-4Δ ARN1* cells

**FIGURE 4:**
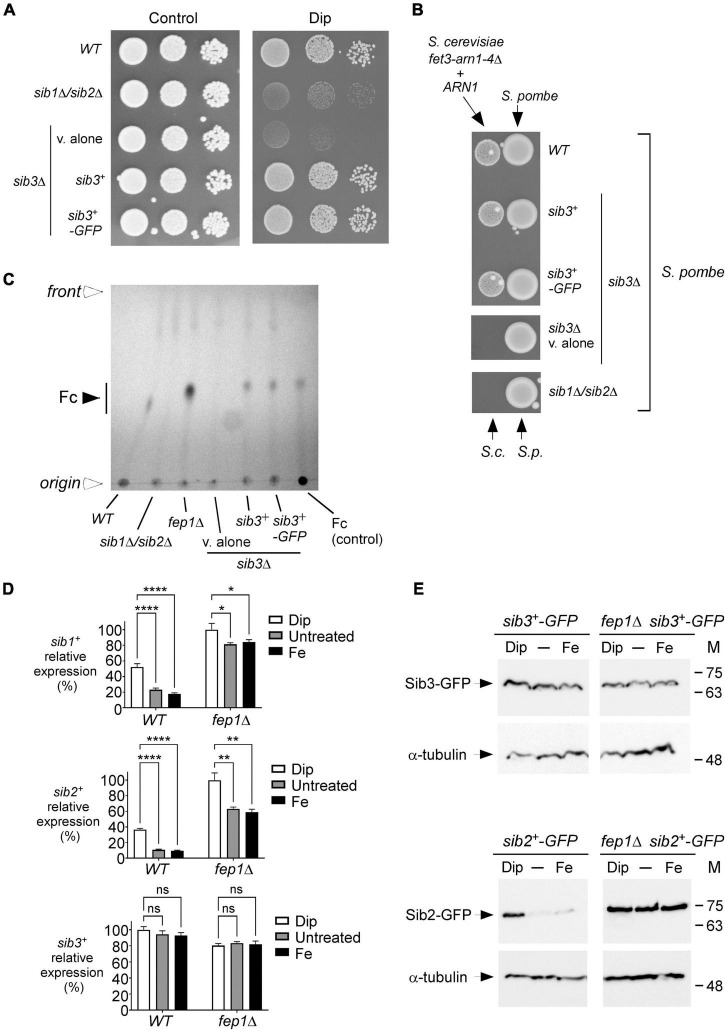
*S. pombe* requires Sib3 to promote Fc-dependent growth of *S. cerevisiae fet3*Δ *arn1-4*Δ cells expressing ARN1. **(A)** Wild-type, *sib1*Δ *sib2*Δ, and *sib3*Δ strains were assayed for their ability to grow on YES medium containing 0 μM (control) or 140 μM Dip. In the case of the *sib3*Δ mutant, an empty plasmid (v. alone), *sib3*^+^, or *sib3^+^-GFP* allele was returned in the strain. Once spotted on the control and iron-starved media, the strains were incubated for 4 days at 30°C, and photographed. **(B)** The indicated *S. pombe* strains described in **(A)** were grown in the presence of iron (10 μM) to an OD_600_ of 1.0. Subsequently, these strains were inoculated (1 × 10^7^ cells/10 μl) (right side of each pair of spots) onto SD^–Cu–Fe^ medium. *S. cerevisiae fet3*Δ *arn1-4*Δ cells expressing *ARN1* were grown to the mid-logarithmic phase, diluted, and spotted (3,000 cells/10 μL) in the vicinity of *S. pombe* strains (left side of each pair of spots). S.c., *S. cerevisiae*; S.p., *S. pombe*. **(C)** The indicated *S. pombe* strains were grown to an OD_600_ of 0.5 in YES medium and incubated in the presence of Dip (100 μM) for 5 h. Total Fc was extracted and analyzed by TLC on silica gel sheets. Commercially purified Fc (15 μg) (control) was loaded as a reference. Solid arrowhead indicates the migration position of Fc, whereas open arrowheads show the origin of sample loading and front of gel migration. **(D)** Shown are expression profiles of *sib1*^+^, *sib2*^+^, and *sib3*^+^ genes. Wild-type (WT) and *fep1*Δ strains were grown in YES medium and were either left untreated or treated with Dip (250 μM) or FeCl_3_ (Fe; 100 μM) for 90 min. Total RNA was prepared from culture aliquots, and steady-state mRNA levels of *sib1*^+^, *sib2*^+^, and *sib3*^+^ were analyzed by RT-qPCR assays. Graphic representations of quantification of three independent RT-qPCR assays. Error bars indicate the standard deviation (± SD; error bars). The asterisks correspond to *p* < 0.05 (*), *p* < 0.01 (**), and *p* < 0.0001 (****) (two-way ANOVA with Tukey’s multiple comparisons test against the indicated strain grown under low-iron conditions), whereas ns stands for not significant. **(E)** A genetic approach was used for allowing homologous integration of the *GFP* coding sequence at the chromosomal loci of *sib2*^+^ and *sib3*^+^, respectively, therefore creating strains containing GFP-tagged *sib2*^+^ and *sib3*^+^ alleles. Subsequently, these two strains were used for disrupting the *fep1*^+^ gene and generating *sib2^+^-GFP fep1*Δ and *sib3^+^-GFP fep1*Δ strains. All four *S. pombe* strains were left untreated (-) or treated with Dip or FeCl_3_ (Fe), as described in **(D)**. Aliquots of whole cell extract preparations were analyzed by immunoblot assays using anti-GFP and anti-α-tubulin antibodies. The positions of the molecular weight standards (in kDa) are indicated on the right side.

According to a proposed pathway for Fc biosynthesis, the first enzymatic step consists of the N^5^ hydroxylation of ornithine by the ornithine-N^5^-oxygenase Sib2 of *S. pombe* to produce N^5^-hydroxyornithine (Haas, 2003, 2014; Haas et al., 2008). This product is predicted to be acetylated by an unknown *S. pombe* N^5^-transacetylase to yield N^5^-acetyl-N^5^-hydroxyornithine (Mercier and Labbé, 2010). On the basis of a prediction inferred from sequence similarity with *Aspergillus fumigatus* transacetylase SidL (Blatzer et al., 2011), the *SPBC17G9.06c* gene encoding a putative acetyltransferase, denoted Sib3, has been suggested to participate in Fc biosynthesis (Mercier and Labbé, 2010). However, its biological role in Fc production has not been demonstrated. Previous studies have shown that *S. pombe* cells lacking Sib1 and Sib2 exhibit severe growth defects in iron-poor media (Mercier and Labbé, 2010; Plante and Labbé, 2019). To test whether an *S. pombe* strain with deletion of the *sib3*^+^ gene phenocopied the inability to grow on iron-deficient media associated with Sib1 and Sib2 deletions, we generated a *sib3*Δ mutant strain. *sib3*Δ cells containing an empty vector were spotted onto an iron-poor medium and compared with *sib1*Δ *sib2*Δ cells. As observed for *sib1*Δ *sib2*Δ cells, *sib3*Δ cells containing an empty vector were unable to grow on a medium supplemented with Dip (140 μM), unlike the wild-type cells, which exhibited growth (Figure 4A). In the case of *sib3*Δ cells containing an untagged *sib3*^+^ or GFP-tagged *sib3*^+^ allele that had been reintegrated, their ability to grow was restored in the presence of Dip (Figure 4A). As controls, the wild-type and mutant strains were equally competent to grow on iron-replete YES medium under non-selective conditions (Figure 4A, left panel).

We next investigated whether the loss of Sib3 affected the ability of the *sib3*Δ mutant strain to promote the growth of the *S. cerevisiae fet3*Δ *arn1-4*Δ *ARN1* strain when they were spotted in the vicinity of each other on SD^–Cu–Fe^ medium. Results showed that *S. pombe sib3*Δ cells containing an empty vector failed to support the growth of the *S. cerevisiae fet3*Δ *arn1-4*Δ *ARN1* strain, unlike the wild-type *S. pombe* strain (Figure 4B). In contrast, this growth defect of *S. cerevisiae fet3*Δ *arn1-4*Δ *ARN1* cells was reversed when they were point-inoculated near *S. pombe sib3*Δ cells expressing an integrated *sib3*^+^ or *sib3^+^-GFP* allele (Figure 4B). As a negative control, the reference *S. pombe sib1*Δ *sib2*Δ strain failed to promote the growth of *S. cerevisiae fet3*Δ *arn1-4*Δ cells expressing *ARN1* (Figure 4B).

We next assessed whether Sib3 is required for Fc biosynthesis. Extracts from the wild-type, *sib1*Δ *sib2*Δ, *fep1*Δ, and *sib3*Δ strains were analyzed by thin-layer chromatography. In contrast to the wild-type or *sib3*Δ strain expressing an untagged *sib3*^+^ or GFP-tagged *sib3*^+^ allele, the *sib3*Δ mutant harboring an empty vector exhibited no detectable Fc signal (Figure 4C). As additional controls, extract preparations from the *sib1*Δ *sib2*Δ mutant strain were devoid of detectable Fc, whereas extracts from the *fep1*Δ mutant exhibited a strong Fc signal because of the lack of complete transcriptional repression of *sib1*^+^ and *sib2*^+^ genes in the absence of Fep1 (Figures 4C,D; Mercier and Labbé, 2010; Plante and Labbé, 2019).

On the basis of the finding that Sib3 is required along with Sib1 and Sib2 for Fc biosynthesis, we tested whether *sib3*^+^ mRNA levels were affected in response to changes in iron concentrations or as a function of the presence or absence of Fep1. Using RT-qPCR assays, we monitored *sib1*^+^, *sib2*^+^, and *sib3*^+^ transcript levels in wild-type (*fep1*^+^) and *fep1*Δ cells grown in either the absence or presence of iron or Dip. The gene expression levels of *sib3*^+^ in Dip- or iron-treated cells were not significantly different compared to the basal levels in untreated cells in both *fep1*^+^ and *fep1*Δ strains (Figure 4D). In accordance with previous results obtained using the wild-type strain (Mercier and Labbé, 2010; Plante and Labbé, 2019), *sib1*^+^ and *sib2*^+^ transcript levels were induced in the presence of Dip. In contrast, their transcript levels were down regulated under basal and iron-replete conditions (Figure 4D). The *sib1*^+^ and *sib2*^+^ mRNA levels were repressed 2.9- and 3.9-fold, respectively, under high-iron conditions relative to the corresponding levels under low-iron conditions (Figure 4D). To validate the predominant role of Fep1 in iron-mediated repression of *sib1*^+^ and *sib2*^+^ transcription, the expression levels of these genes were derepressed in both untreated and iron-treated *fep1*Δ cells (Figure 4D). Although the levels of derepression were not as high as that under iron-limiting conditions, they were considerably higher (4.7- and 6.2-fold, respectively) than that observed under the same iron-replete conditions in the wild-type strain (Figure 4D).

To ascertain whether the steady-state protein levels of Sib2-GFP and Sib3-GFP were consistent with that of *sib2^+^-GFP* and *sib3^+^-GFP* transcripts, we created strains in which the *GFP* coding sequence was integrated at the chromosomal loci of *sib2*^+^ and *sib3*^+^ in the wild-type and *fep1*Δ mutant strains. These strains were grown to the logarithmic phase and left untreated or treated with Dip (250 μM) or FeCl_3_ (100 μM) for 90 min. Whole cell extracts were prepared and analyzed by immunoblotting. The results showed that Sib3-GFP steady-state levels were constitutively expressed in both the wild-type and *fep1*Δ strains, irrespective of the cellular iron status (Figure 4E). In the case of steady-state protein levels of Sib2-GFP, results showed that they were elevated in the presence of Dip but low in untreated or iron-treated wild-type cells. In the case of *fep1*Δ cells expressing *sib2^+^-GFP*, results showed that Sib2-GFP protein levels remained elevated and nearly unchanged under all experimental conditions (Figure 4E). Taken together, these results indicate that Sib3 is constitutively expressed and its presence is required for the Fc production, which could be utilized as an iron source by *S. cerevisiae fet3*Δ *arn1-4*Δ *ARN1* cells.

### Sib1, Sib2, and Sib3 proteins exhibit a common subcellular localization under low-iron conditions

**FIGURE 5:**
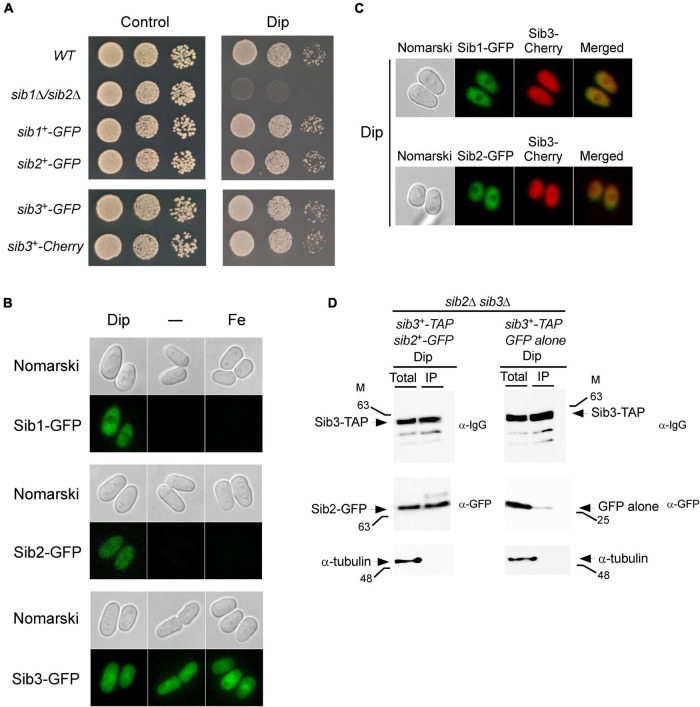
Localization of Sib1, Sib2 and Sib3 proteins in *S. pombe*. **(A)** Strains in which the *GFP* coding sequence was directly integrated at the chromosomal locus of *sib1*^+^, *sib2*^+^, or *sib3*^+^ were created and termed *sib1*^+^-GFP, *sib2*^+^-GFP, and *sib3*^+^-GFP strains, respectively. In the case of *sib3*^+^, an identical approach was performed using the Cherry coding sequence, thereby creating the *sib3*^+^-Cherry strain. Wild-type (WT), *sib1*Δ *sib2*Δ, *sib1*^+^-GFP, *sib2*^+^-GFP, *sib3*^+^-GFP, and *sib3*^+^-Cherry strains were grown to the logarithmic phase under non-selective growth conditions and spotted in serial dilutions onto YES medium without Dip (control) or with Dip (140 μM). **(B)** Strains expressing *sib1^+^-GFP*, *sib2^+^-GFP*, and *sib3^+^-GFP* alleles under the control of their own promoters were untreated (-) or treated with Dip (250 μM) or FeCl_3_ (100 μM) for 90 min. Cells were analyzed by fluorescence microscopy for the presence of GFP-dependent fluorescence signals (*bottom of each pair of panels*). Nomarski optics (*top of each pair of panels*) was used for examining cell morphologies. **(C)** Strains co-expressing Sib3-Cherry in combination with Sib1-GFP or Sib2-GFP were incubated in the presence of Dip (250 μM) for 90 min. Fluorescence signals of Sib3-Cherry co-expressed with Sib1-GFP or Sib2-GFP were observed by fluorescence microscopy (*middle panels*). Merged images of co-expressed fluorescent proteins are shown in the *far right panels*. Cell morphology was examined by Nomarski optics in the *far left panels*. Results of microscopy are representative of five independent experiments. **(D)** *sib2*Δ *sib3*Δ cells co-expressing GFP-tagged Sib2 and TAP-tagged Sib3 or GFP alone and TAP-tagged Sib3 were grown to the mid-logarithmic phase and incubated in the presence of Dip (250 μM) for 90 min. Whole cell extracts (Total) were incubated with IgG-Sepharose beads. The immunoprecipitated fractions (IP) were analyzed by immunoblot assays using anti-mouse IgG (α-IgG), anti-GFP (α-GFP) and anti-α-tubulin antibodies. Aliquots of whole cell extracts (Total) were probed with the same antibodies to ascertain the presence of epitope-tagged proteins prior to incubation with beads. As an additional control, aliquots of total cell lysates and bound fractions were assayed using an anti-α-tubulin antibody. The positions of the molecular weight standards (in kDa) are indicated on the left and right sides of the panels.

The finding that Sib1, Sib2, and Sib3 participated in Fc biosynthesis suggested that these proteins share a common subcellular localization when they are active, especially under low-iron conditions. To ascertain the localization of Sib1, Sib2, and Sib3 proteins in *S. pombe*, functional GFP-tagged *sib1*^+^, *sib2*^+^, and *sib3*^+^ alleles were created. In the case of *sib3*^+^, its GFP- or Cherry-tagged version functionally complemented the growth deficiency of a *sib3*Δ strain on iron-depleted media in a manner indistinguishable from the untagged *sib3*^+^ allele (Figures 4A, 5A). For *sib1*^+^ and *sib2*^+^, two *S. pombe* strains were used in which the *GFP* coding sequence was directly integrated at these chromosomal loci. Cultures of the strains expressing *sib1^+^-GFP* and *sib2^+^-GFP* were grown, and a serial dilution series of each culture was analyzed to test whether they restored growth defects in a medium supplemented with Dip compared to the wild-type strain for which growth was observed (Figure 5A). As expected, strains harboring GFP-tagged *sib1*^+^ and *sib2*^+^ alleles regained the ability to grow on medium containing Dip (Figure 5A). In contrast, the *sib1*Δ *sib2*Δ mutant strain was unable to grow on the medium supplemented with Dip, unlike the wild-type strain (Figure 5A).

Next, we examined the cellular localization of GFP-tagged Sib1, Sib2, and Sib3 proteins in cells that had been incubated in the absence or presence of Dip or FeCl_3_. Under low-iron conditions, fluorescence microscopy showed that Sib1-GFP and Sib2-GFP fluorescent signals were primarily observed throughout the cytoplasm and that they appeared to be largely absent in the nucleus (Figure 5B). Consistent with the iron-dependent regulated expression of *sib1*^+^ and *sib2*^+^, Sib1-GFP and Sib2-GFP fluorescence signals were lost when the cells were untreated (basal conditions) or treated with high concentrations of iron (100 μM FeCl_3_) (Figure 5B). The green fluorescence signal associated with Sib3-GFP was pancellular; it was detected throughout the cytoplasm and nucleus. Furthermore, the Sib3-GFP-associated fluorescent signal remained unchanged under basal, iron-starved, and iron-replete conditions (Figure 5B).

To further examine the cellular localization of fluorescent fusion proteins when co-expressed in cells, experiments were set by integrating functional *sib1^+^-GFP* and *sib3^+^-Cherry* or *sib2^+^-GFP* and *sib3^+^-Cherry* alleles into *sib1*Δ *sib3*Δ, *sib2*Δ *sib3*Δ, or *sib1*Δ *sib2*Δ *sib3*Δ cells. For simplicity, we have presented the results obtained using the *sib1*Δ *sib2*Δ *sib3*Δ triple mutant strain in which the indicated alleles were returned because identical results were observed using the *sib1*Δ *sib3*Δ or *sib2*Δ *sib3*Δ strain prior to the reintroduction of *sib1^+^-GFP* and *sib3^+^-Cherry* or *sib2^+^-GFP* and *sib3^+^-Cherry* alleles. Following the growth of cells under low-iron conditions, fluorescence microscopy analysis showed that co-expression of Sib1-GFP and Sib3-Cherry or Sib2-GFP and Sib3-Cherry exhibited identical patterns of fluorescent signals compared to that observed when each of the fluorescent proteins was expressed separately in iron-starved cells (Figure 5C). Sib1-GFP and Sib2-GFP fluorescent proteins were primarily localized in the cytosol, whereas the Sib3-Cherry fluorescent signal displayed a pancellular distribution (Figure 5C). Taken together, these results indicate that all three proteins can be found in the cytosol, where they participate in Fc biosynthesis.

Considering that Sib2 and Sib3 represent the first two enzymes of the pathway that are required for Fc biosynthesis, and the observation that they exhibited a common subcellular localization suggested that these proteins could interact with one another. To test whether Sib2 and Sib3 are interacting partners, *S. pombe sib2*Δ *sib3*Δ mutant cells were used for co-expressing *sib3^+^-TAP* and *sib2^+^-GFP* or *sib3^+^-TAP* and *GFP* alleles. *S. pombe* cells were grown in a medium containing Dip (250 μM). Whole-cell extracts were prepared and subsequently incubated in the presence of IgG-Sepharose beads that selectively bound TAP-tagged Sib3. The results showed that Sib3-TAP was retained on the beads and detected in the immunoprecipitated fraction (IP) (Figure 5D). Furthermore, the IP contained Sib2-GFP, which interacted with Sib3-TAP (Figure 5D). Interaction between Sib3-TAP and GFP alone was not observed in pull-down experiments, unlike the interaction between Sib3-TAP and Sib2-GFP (Figure 5D). To assess the specificity of the pull-down experiments, whole-cell extracts (Total) and bound fractions (IP) were analyzed by immunoblotting using an antibody directed against α-tubulin. The results showed that α-tubulin was present in whole cell extracts but not in IPs (Figure 5D). Taken together, these results revealed that Sib2-GFP and Sib3-TAP interact with each other to form a protein complex that is co-immunoprecipitated from whole-cell extracts isolated from iron-starved cells.

## Discussion

In this study, we established experimental culture conditions in which *S. cerevisiae* cells that are unable to synthesize Fc took advantage of Fc secreted from *S. pombe* cells to survive under iron deficiency. Considering that this exchange of Fc was unidirectional, with donor *S. pombe* cells providing Fc to recipient *S. cerevisiae* cells, the interaction was classified as one-way cross-feeding. Fc serves as an essential metabolite required for the cellular growth of *S. cerevisiae* as a sole source of iron. Although previous studies have reported that siderophores can participate in one-way cross-feeding between different bacterial species (Joshi et al., 2006; Galet et al., 2015; Butaitë et al., 2017; D’Souza et al., 2018), such cross-feeding interactions are much less known between different fungal species. A previous study suggested that Fc secreted by *S. pombe* allows conidial germination of an *Aspergillus nidulans sidA*Δ strain deficient in siderophore synthesis (Schrettl et al., 2004). However, the latter study only used a wild-type *S. pombe* strain without further characterization of the genes and proteins required for establishing one-way cross-feeding between the two yeast species. Another study showed that *Paracoccidiodes lutzii* and *P. brasiliensis* yeasts could donate siderophores to a non-producer *A. nidulans sidA*Δ mutant (Silva-Bailão et al., 2014). In *Paracoccidiodes* yeasts, the identity of cellular components involved in one-way cross-feeding with *A. nidulans sidA*Δ cells remains unknown. Thus, in fungal species, the nature of molecules and mechanisms whereby cross-feeding occurs remain poorly defined.

To identify the *S. pombe* genes required for Fc production, which can affect the survival of a neighboring yeast, we set experimental conditions in which *S. cerevisiae* obligately utilized *S. pombe* Fc to survive. As expected, we found that disruption of the *sib1*^+^ and *sib2*^+^ genes of *S. pombe* blocked Fc biosynthesis, thus preventing Fc-dependent growth of *S. cerevisiae* cells. To identify a putative transacetylase involved in Fc biosynthesis, we initially searched the entire set of genetic information of the *S. pombe* database. Analysis of genomic DNA sequences revealed 38 genes that encode known or putative members of the GCN5-related N-acetyltransferase (GNAT) protein family. This family of proteins includes functionally diverse enzymes that catalyze the transfer of an acetyl group from acetyl-CoA to the primary amine of different acceptor substrates. Among them, only one gene (*SPBC17G9.06c*, denoted *sib3*^+^) contains a pfam10331/AlcB domain, which is typically found in siderophore-biosynthetic transacetylase enzymes (de Lorenzo et al., 1986; Card et al., 2005; Challis, 2005; Blatzer et al., 2011). Enzymes that catalyze the transfer of an acetyl group from acetyl-CoA during the biosynthesis of siderophores include *Bordetella bronchiseptica* AlcB, *E. coli* IucB, *Mycobacterium tuberculosis* Rv1347c, and *Aspergillus fumigatus* SidL (de Lorenzo et al., 1986; Card et al., 2005; Challis, 2005; Blatzer et al., 2011).

Inactivation of N^5^-hydroxyornithine acetyl-CoA-N^5^-transacetylase SidL (*sidL*Δ) prevents ferricrocin biosynthesis under iron-replete conditions (Blatzer et al., 2011). However, a *sidL*Δ mutant is still able to produce ferricrocin under low-iron conditions, suggesting the existence of a second uncharacterized N^5^-hydroxyornithine acetyl-CoA-N^5^-transacetylase that is induced to compensate for the loss of SidL (*sidL*Δ) under iron-poor conditions (Blatzer et al., 2011). Deletion of *S. pombe* Sib3 blocked Fc biosynthesis irrespective of cellular iron status. Moreover, an *S. pombe sib3*Δ mutant failed to provide Fc to *S. cerevisiae fet3*Δ *arn1-4*Δ *ARN1* cells when the two yeast species were spotted in close proximity, resulting in growth defects in the case of *S. cerevisiae fet3*Δ *arn1-4*Δ *ARN1* cells. In contrast to *A. fumigatus*, *S. pombe* does not possess a second N^5^-hydroxyornithine acetyl-CoA-N^5^-transacetylase because inactivation of *sib3*^+^ (*sib3*Δ) results in a complete inability to produce Fc under both iron-replete and iron-depleted conditions.

In general, the expression of fungal genes encoding siderophore biosynthetic enzymes is transcriptionally repressed in response to high iron concentrations (Haas, 2003, 2014; Haas et al., 2008). In contrast, the transcript levels of these genes are generally induced under iron-starvation conditions. A few exceptions include the *npgA* and *sidL* genes in *A. nidulans* that are constitutively expressed (Oberegger et al., 2003; Blatzer et al., 2011). Similar to *A. nidulans npgA* and *sidL*, *sib3*^+^ is constitutively expressed in *S. pombe* under basal, iron-starved, and iron-replete conditions. The iron-dependent downregulation of fungal iron-regulated siderophore biosynthetic genes is primarily mediated by iron-regulatory GATA-type transcription factors, including SreA in *A. nidulans* and *A. fumigatus*, URBS1 in *Ustilago maydis*, and Fep1 in *S. pombe* (Haas et al., 2008; Brault et al., 2015; Hortschansky et al., 2017). However, Sib3 being a constitutively expressed gene, the mRNA and protein levels of Sib3 did not vary significantly with respect to either the presence or absence of Fep1, confirming its non-canonical expression profile compared to that of classical iron regulon genes.

The localization of *S. pombe* Sib3 was observed throughout the cells, suggesting that it is localized to both the cytosol and nucleus. In contrast, the Sib1 and Sib2 proteins were primarily cytosolic. Because the common cellular compartment of Sib1, Sib2, and Sib3 is the cytosol, Fc biosynthesis most likely occurs in the cytoplasmic compartment, as it has been proposed for ferricrocin biosynthesis in *A. fumigatus* (Haas, 2014). For Fc biosynthesis, Sib2 is required for the conversion of ornithine to N^5^-hydroxyornithine. The results presented here reveal that Sib2 and Sib3 co-localize in the cytosol and interact with one another. One can envision that the step involving Sib2-dependent conversion of ornithine possibly includes an additional mechanism that favors the simultaneous recruitment of Sib3 to facilitate the next sequential reaction, which consists of the acetylation of N^5^-hydroxyornithine. This would favor the first two sequential reactions, allowing efficient accumulation of N^5^-acetyl-N^5^-hydroxyornithine that needs to be combined with three glycine residues to assemble the finished Fc product through the action of Sib1.

As previously reported in the case of *S. cerevisiae* Arn1 (Kim et al., 2002), our results showed that the protein primarily localizes to the endosomal compartment under basal growth conditions in a Fc-free medium. When *S. cerevisiae* cells are exposed to low concentrations of Fc under low-iron conditions, Arn1 undergoes re-localization from the endosomal compartment to the plasma membrane (Kim et al., 2002, 2005). Our results showed that the localization of *S. pombe* Str1 moved away from an intracellular compartment to occupy the periphery of the cell when cells had been treated with Dip or Dip plus Fc. At this point, however, it is unclear how Str1 re-localized to the cell surface in the presence of Fc under low-iron conditions. In the case of *S. cerevisiae* Arn1, studies have shown that an extracytosolic carboxyl-terminal domain of the protein is required for its cycling between the plasma membrane and endosomal compartments, where uptake of Fc occurs (Kim et al., 2005). At this stage, although the carboxyl-terminal region of Str1 contains conserved amino acid residues with the Fc-responsive extracytosolic loop of Arn1, the identification of a specific Fc receptor domain of Str1 that may control its trafficking will require additional studies.

Following Fc biosynthesis, one can envision the existence of transporter(s) for Fc secretion. Fc secretion is believed to be a necessary step for releasing Fc into the environment before the reuptake of Fc-bound iron by *S. pombe* cells. As shown in this study, the secreted Fc could be recovered and utilized by *S. cerevisiae* cells in the vicinity of *S. pombe* cells. Key cell-surface membrane proteins have been identified for the secretion of enterobactin from *E. coli* cells into the extracellular environment (Wilson et al., 2016). First, enterobactin is exported from the cytosol to the periplasm by EntS, a MFS-type transporter. Subsequently, the efflux of enterobactin across the outer membrane involves the concerted action of a resistance/nodulated/cell division (RND)-class transporter (AcrB, AcrD, or MdtABC) and the protein channel TolC (Horiyama and Nishino, 2014; Wilson et al., 2016). Considering the complexity of the siderophore efflux system in *E. coli*, it is not surprising that little is known regarding siderophore secretion in fungi. In *Aspergillus fumigatus*, fusarinine C (FsC) and triacetylfusarinine C (TAFC) siderophores are produced and secreted into the external environment (Schrettl et al., 2007). Although the proteins involved in their biosynthesis have been identified, the transporters for the secretion of FsC and TAFC have not yet been identified. Further investigation will be necessary to decipher the precise mechanisms by which *S. pombe* secretes Fc into the external milieu.

## Data availability statement

The original contributions presented in this study are included in the article/supplementary material, further inquiries can be directed to the corresponding author.

## Author contributions

AB, BM, and SL: methodology, validation, formal analysis, investigation, review and editing, and resources. AB and SL: conceptualization, writing—original draft preparation. SL: supervision, project administration, and funding acquisition. All authors have read, reviewed and approved the final version of the manuscript.

## Conflict of interest

The authors declare that the research was conducted in the absence of any commercial or financial relationships that could be construed as a potential conflict of interest.

## Publisher’s note

All claims expressed in this article are solely those of the authors and do not necessarily represent those of their affiliated organizations, or those of the publisher, the editors and the reviewers. Any product that may be evaluated in this article, or claim that may be made by its manufacturer, is not guaranteed or endorsed by the publisher.

## Footnotes

1 We use the standard nomenclature for *S. cerevisiae* and *S. pombe* genes and proteins. *S. cerevisiae* wild-type genes are capitalized and italicized (e.g., *ARN1*). *S. pombe* wild-type genes are designated as three italicized lowercase letters, with a superscripted “+” at the end (e.g., *str1*^+^). *S. cerevisiae* and *S. pombe* proteins are written in plain text with one capital letter, followed by two lowercase letters and a number (e.g., Arn1 and Str1).

## Abbreviations

bp: base pair(s)
Dip, 2: 2′-dipyridyl
EMM: Edinburgh minimal medium
Fc: ferrichrome
FsC: fusarinine
TAFC: triacetylfusarinine C
GFP: green fluorescent protein
GNAT: GCN5-related N-acetyltransferase
MFS: major facilitator superfamily
NRPS: non-ribosomal peptide synthetase
ORF: open reading frame
PCR: polymerase chain reaction
RND: resistance/nodulated/cell division
RT-PCR: reverse transcription PCR
RT-qPCR: real-time quantitative reverse transcription PCR
SC: synthetic complete medium
SDS: sodium dodecyl sulfate
TLC: thin-layer chromatography
WT: wild-type
YES: yeast extract plus supplements.
